# Cost-effectiveness analysis of eltrombopag, hetrombopag and romiplostim for second-line treatment of primary immune thrombocytopenia in China

**DOI:** 10.3389/fmed.2026.1916651

**Published:** 2026-08-24

**Authors:** Xi Chen, Yadi Liu, Jianping Zhang, Rong Xu, Cheng Guo

**Affiliations:** Department of Pharmacy, Shanghai Sixth People’s Hospital Affiliated to Shanghai Jiao Tong University School of Medicine, Shanghai, China

**Keywords:** primary immune thrombocytopenia, eltrombopag, romiplostim, hetrombopag, cost-effectiveness, treatment-sequence Markov model

## Abstract

**Background:**

Primary immune thrombocytopenia (ITP) is a chronic autoimmune bleeding disorder requiring long-term second-line treatment in patients refractory to corticosteroids and immunoglobulins. In China, eltrombopag, hetrombopag, and romiplostim are all available after national reimbursement negotiations, but comparative economic evidence to inform optimal therapeutic selection remains limited. This study aimed to evaluate the cost-effectiveness of eltrombopag, hetrombopag, and romiplostim as second-line treatments for primary ITP in China from the healthcare system perspective.

**Methods:**

A lifetime Markov model was developed to estimate costs and quality-adjusted life-years (QALYs) associated with each treatment strategy. Clinical inputs were derived from phase III randomized controlled trials in Chinese populations and supplemented with indirect treatment comparisons where head-to-head evidence was unavailable. Long-term disease progression and relapse were extrapolated using published literature and clinical assumptions consistent with ITP disease course. Costs were obtained from domestic medical databases, and utility values were sourced from published studies. Both deterministic and probabilistic sensitivity analyses were conducted to assess parameter uncertainty. Scenario analyses were performed to explore the impact of different time horizons on model outcomes.

**Results:**

Compared with eltrombopag, hetrombopag and romiplostim yielded incremental health gains of 0.02 QALYs and 0.05 QALYs with incremental costs of 41,329.52 CNY and 99,009.66 CNY, generating ICERs of 2,484,284.17 CNY/QALY and 2,077,773.05 CNY/QALY, respectively. When compared with hetrombopag, romiplostim was associated with an ICER of 1,859,724.27 CNY/QALY. Deterministic sensitivity analysis identified treatment response rates, drug acquisition costs and discount rate as key drivers of model outcomes. Probability sensitivity analysis revealed that at a willingness-to-pay threshold of 2 times GDP per capita, eltrombopag regimen demonstrated the highest probability of being cost-effective among the three strategies. Results of scenario analyses verified the robustness of base-case results.

**Conclusion:**

Eltrombopag was likely to be the most cost-effective second-line treatment option for ITP compared with hetrombopag and romiplostim. Our findings provide economic evidence to support decision-making regarding the use of TPO-RAs in China.

## Introduction

Primary immune thrombocytopenia (ITP) is a bleeding disorder characterized by reduced platelet count and impaired coagulation function (1), accounting for one-third of all hemorrhagic diseases (2). Epidemiological data from multiple provincial surveys in China suggest that the annual incidence of ITP in the Chinese population is approximately 3–7 per 100,000 (3–5). If platelet counts decrease persistently below safe levels, patients are at risk of life-threatening bleeding events (6), including severe visceral and intracranial hemorrhage. Mortality risk of patients with ITP was approximately 1.5 times higher than that of the general population (7). Fatigue symptoms, bleeding episodes along with anxiety about platelet levels significantly diminished health-related quality of life (HRQoL) of patients (8). Meanwhile, ITP imposes a heavy economic burden on society as data analyzed by Weycker et al. (9) showed that during 2010–2016, the average annual total medical cost of newly diagnosed ITP was $21,290 and the overall annual economic burden caused by ITP exceeded $400 million in the United States. A study based on Chinese real-world data from six tertiary hospitals estimated that per capita direct medical costs associated with ITP was over ¥46,000, in which hospitalization costs comprised up to 92.2 and 77.4% for drug costs (10).

The treatment of ITP is generally tailored to individuals depending on the symptoms, with the goal of elevating platelet counts and reducing bleeding events while ensuring minimization of adverse events (AEs) (11). Glucocorticoids (including high-dose dexamethasone and prednisone) or intravenous immunoglobulin (IVIg) are preferred for first-line treatment of ITP (11). However, a considerable proportion of adult patients fail to achieve sustained responses after first-line therapy and subsequently require second-line therapies (12). Current expert recommendations and reviews have emphasized that treatment selection for adults with ITP should be individualized according to disease characteristics, bleeding risk, prior treatment response, and patient preferences (12, 13). Available second-line options include thrombopoietin receptor agonists (TPO-RAs), rituximab, splenectomy, and other immunomodulatory therapies (12, 13). Among these therapies, TPO-RAs have become important treatment options for adults with persistent or chronic ITP because of their ability to stimulate platelet production and improve platelet responses (13). According to American Society Hematology 2019 guidelines for immune thrombocytopenia, a TPO-RA was recommended rather than rituximab or splenectomy for adults who had been diagnosed with ITP for more than 3 months and underwent failed treatment of corticosteroids (12) due to limitations of rituximab and splenectomy (14). Although most patients with ITP do not experience life-threatening bleeding, critical bleeding represents a severe complication requiring urgent intervention. Recent systematic evidence indicates that management strategies for critical bleeding commonly involve corticosteroids, IVIg, platelet transfusion, and other rescue approaches; however, robust evidence to guide optimal management remains scarce (15).

Approved TPO-RAs for the second-line treatment of ITP in China include eltrombopag, hetrombopag and romiplostim. Eltrombopag is an oral small-molecule nonpeptide TPO-RA which stimulates the proliferation and differentiation of megakaryocytes, generating increased platelet production (16, 17). A double-blind phase III randomized controlled trial (18) which compared the efficacy of eltrombopag with placebo demonstrated significant platelet elevation and longer duration of response in the eltrombopag group. Hetrombopag acts similarly as eltrombopag and showed better performance, bringing about better response rates with the platelet count level higher than 50 × 10^9^/L compared with placebo (19). Romiplostim, a peptibody TPO-RA by subcutaneous injection, exhibited satisfying platelet production and time of response duration as reported in the randomized controlled trial (20).

Although novel mechanism-based agents, including spleen tyrosine kinase (SYK) inhibitors and Bruton’s tyrosine kinase (BTK) inhibitors, have shown promising efficacy in international studies (11), these therapies have not yet been approved for marketing in China and lack domestic pricing data; similarly, efgartigimod has not been approved for the indication of ITP. Therefore, these therapeutic classes were not incorporated into the analytical framework.

The aforementioned three TPO-RAs had been approved for treating adult patients who had an insufficient response to corticosteroids or IVIg, and were included in the National Drug Reimbursement List (NDRL) through national reimbursement negotiation. There is a lack of economic evidence which supports the selection of second-line treatment for ITP in China. Accordingly, this study aimed to evaluate the cost-effectiveness of eltrombopag, hetrombopag and romiplostim as second-line treatments for ITP in a gesture to better inform clinical decisions in ITP.

## Materials and methods

### Target population

The target population was aligned with the indication covered by NRDL for TPO-RAs in China- adult ITP patients who have previously received corticosteroids and IVIg with insufficient response. Based on the inclusion criteria of the pivotal TPO-RA trials (18–20), insufficient response was defined as: (i) a platelet count < 30 × 10^9^/L at screening; (ii) previous treatment administered at adequate doses, with maintenance immunosuppressive therapy (e.g., corticosteroids) stable for ≥ 30 days and any rescue therapy (e.g., IVIg) completed ≥ 2 weeks before baseline assessment; and (iii) failure determined during the screening period based on platelet counts, indicating loss of durable response to prior therapy. It was assumed that patients’ average age was 42 years and the male proportion was 27.9%, derived from the pooled baseline demographics of the three pivotal RCTs (18–20). Referring to the recommendation from National Healthcare Security Administration (NHSA), body weight of the population was assumed to be 60 kg and body surface area was 1.6 m^2^.

### Interventions and regimens

**FIGURE 1:**
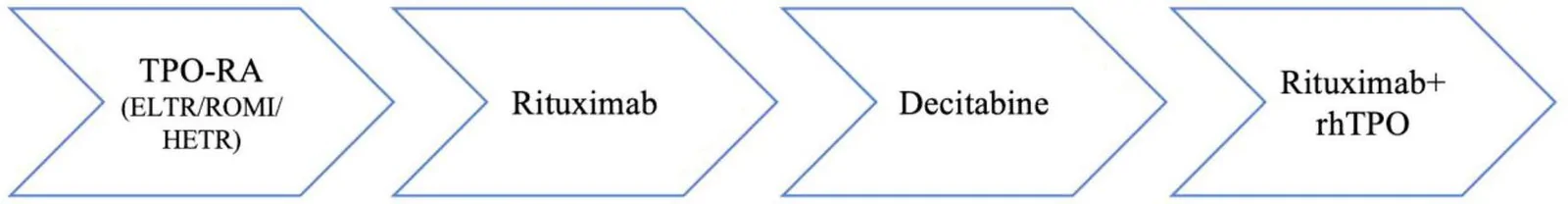
Treatment sequence for Primary Immune Thrombocytopenia. TPO-RA, thrombopoietin receptor agonist; ELTR, eltrombopag; ROMI, romiplostim; HETR, hetrombopag; rhTPO, recombinant human thrombopoietin.

According to the 2025 Chinese guideline for the diagnosis and management of ITP (11), second-line treatment following failure or relapse of first-line therapy emphasizes individualized therapeutic selection, with TPO-RAs, rituximab, and other immunomodulatory strategies constituting the mainstay options. The study pathway was defined based on currently available and guideline-recommended regimens, including TPO-RAs, rituximab, decitabine, and rituximab combined with rhTPO, thereby ensuring both methodological rigor and relevance to the Chinese healthcare setting. Those maintaining a response continued treatment with eltrombopag, hetrombopag, or romiplostim until treatment failure. A relapse event triggered progression to the next modeled treatment line, comprising options such as rituximab, decitabine and rituximab in combination with rhTPO, as outlined in Figure 1.

Dosing regimens for eltrombopag and hetrombopag were 50 and 5 mg per day, respectively. Romiplostim was injected subcutaneously by 3 μg/kg every week, consistent with the most commonly used starting dose in published studies (21, 22) and closely aligned with the weekly average dose (2.97 μg/kg) reported in the pivotal RCT (20). Blood counts and liver function were monitored regularly during treatment. Upon relapse, rituximab was administered intravenously at 375 mg/m^2^ once weekly for 4 weeks. If patients failed to respond to rituximab, treatment was switched to decitabine, administered intravenously daily at 3.5 mg/m^2^ for 3 consecutive days per cycle, for a total of 3 cycles. In cases of further relapse, a combination regimen was administered which consisted of rituximab (100 mg/m^2^ weekly for 4 weeks) plus rhTPO (300 U/kg daily for 14 days).

In the event of life-threatening hemorrhage or the need for emergency surgery, rescue therapy was administered to rapidly elevate platelet counts to a safe level. The adopted rescue regimens comprised intravenous immunoglobulin (IVIg) of 1 g/kg for 1 day, intravenous methylprednisolone 1,000 mg daily for 3 days, subcutaneous rhTPO at 300 U/kg, and platelet transfusion.

### Model design

**FIGURE 2:**
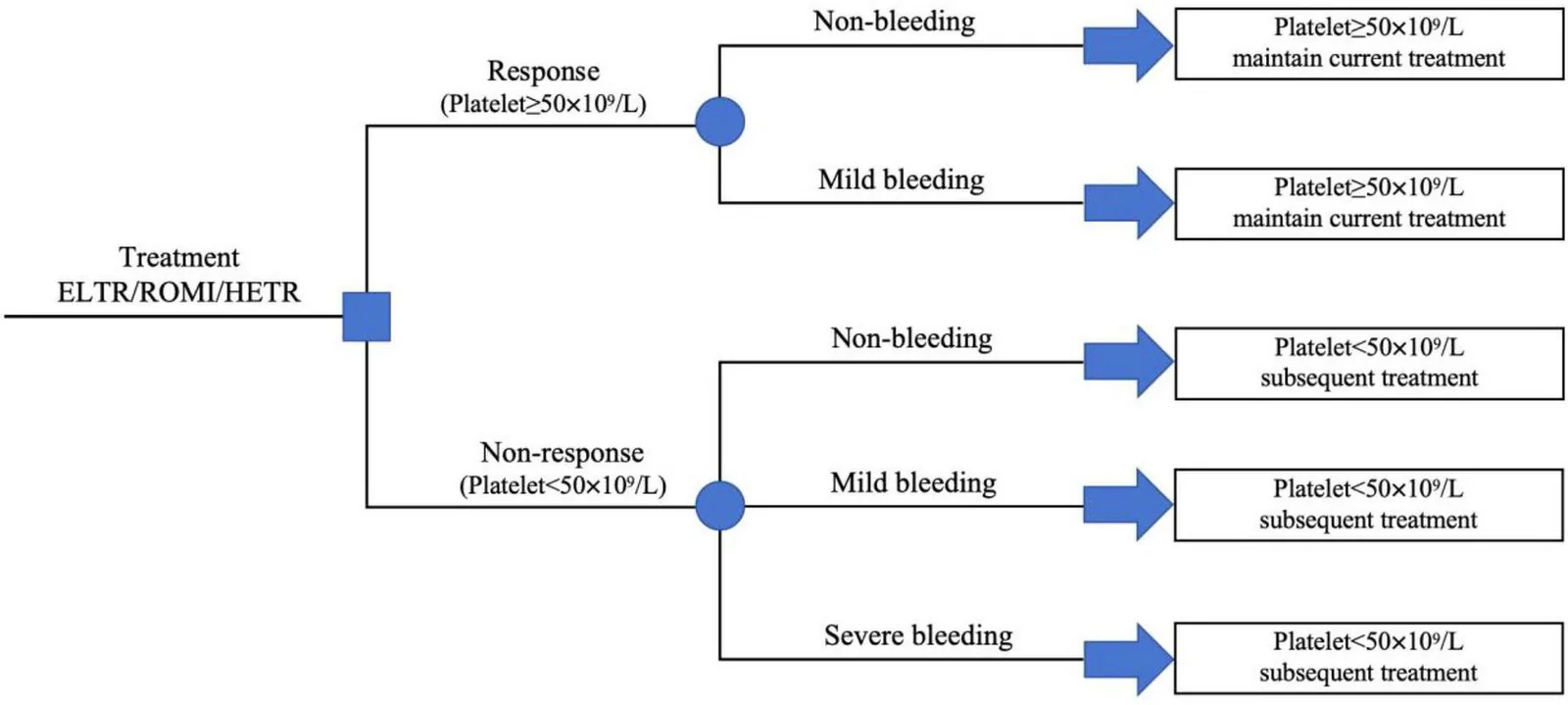
A Schematic diagram of embedded decision tree. ELTR, eltrombopag; ROMI, romiplostim; HETR, hetrombopag.

**FIGURE 3:**
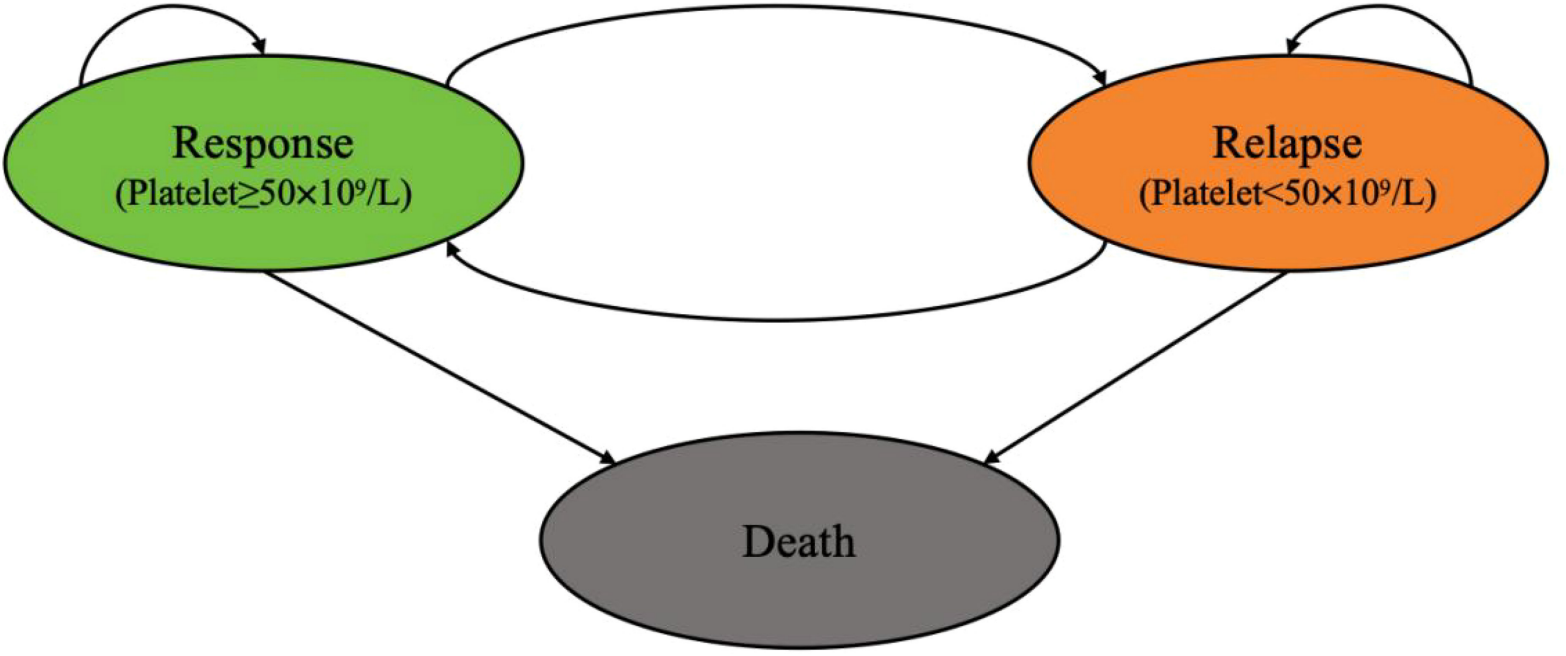
Markov model overview.

A treatment-sequence Markov model with an embedded decision tree was constructed to estimate long-term costs and health outcomes. A lifetime (30 years) time horizon was set to reflect the chronic course of ITP. The model used 4 week cycles corresponding to the treatment dosing schedules. The model structure, as illustrated in Figures 2, 3, incorporated three health states: response (platelet count ≥ 50 × 10^9^/L), relapse (platelet count < 50 × 10^9^/L), and death. In the model, platelet response served as the basis for determining treatment effectiveness, the progression through the therapeutic pathway and risk of bleeding. Within each cycle, patients in response remained in that state and received particular treatments. Once relapse events occurred, patients transitioned to receive the subsequent treatment regimen, namely, rituximab, decitabine and rituximab+rhTPO. The willingness-to-pay (WTP) threshold was established at 2-time GDP per capita in 2025. The principal outputs of the model comprised total costs, quality-adjusted life-years (QALYs), and incremental cost-effectiveness ratios (ICERs), all discounted at a rate of 4.5% according to the current recommendation of China Guidelines for Pharmacoeconomic Evaluations (23). Furthermore, a half-cycle correction was applied to both cost and effectiveness estimates.

### Clinical inputs

Key clinical effectiveness inputs in the model included response rates and relapse rates. Since there were no clinical trials directly compared the efficacy of three study drugs, an indirect comparison of pivotal RCTs (18–20) was performed to obtain response rates for each regimen. The information of key RCTs were outlined in Supplementary Table S1.

An adjusted indirect comparison was performed using placebo as the common comparator based on key RCTs (18–20). Odds ratios (ORs) for response versus placebo were estimated for eltrombopag, hetrombopag, and romiplostim using the netmeta package in R interpreted as a placebo-anchored indirect comparison. A pooled placebo response rate was calculated by combining responders and total patients across the placebo arms of the included trials. Absolute response rates for each active treatment were then derived using the formula below, in which P_*drug*_ refers to the pooled placebo response rate.


$$P_{d\,r\,u\,g}=\frac{P_{p\,l\,a\,c\,e\,b\,o}\times O\,R_{d\,r\,u\,g,p\,l\,a\,c\,e\,b\,o}}{1-P_{p\,l\,a\,c\,e\,b\,o}+P_{p\,l\,a\,c\,e\,b\,o}\times O\,R_{d\,r\,u\,g,p\,l\,a\,c\,e\,b\,o}}$$

Indirect comparison results of response rates for study drugs were displayed in Table 1, Supplementary Figure S1 and Supplementary Table S2. Due to a lack of long-term efficacy evidence of study regimens for Chinese patients, the model was populated with constant response rates.

**TABLE 1:** Summary of model inputs.

Model inputs	Deterministic	Minimum	Maximum	Distribution	Source
Response rate					
Eltrombopag	0.53	0.42	0.63	Beta	(18)
Hetrombopag	0.60	0.48	0.72	Beta	Calculated based on indirect comparison
Romiplostim	0.70	0.56	0.84	Beta	Calculated based on indirect comparison
Relapse rate					
Eltrombopag	0.011	0.009	0.013	Beta	(24)
Hetrombopag	0.011	0.009	0.013	Beta	Assumed to be equal to eltrombopag
Romiplostim	0.007	0.006	0.008	Beta	(24)
Bleeding rate					
Response state without bleeding	0.87	0.70	1.05	Beta	(28)
Response state with mild bleeding	0.13	0.10	0.15	Beta	(28)
Relapse state without bleeding	0.56	0.44	0.67	Beta	(28)
Relapse state with mild bleeding	0.41	0.33	0.49	Beta	(28)
Relapse state with severe bleeding	0.04	0.03	0.04	Beta	(28)
Rescue rate					
Eltrombopag	0.09	0.07	0.10	Beta	(18)
Hetrombopag	0.10	0.06	0.15	Beta	(19)
Romiplostim	0.01	0.01	0.02	Beta	(20)
Relative risk of death in relapse state compared with response state	1.50	1.10	2.20	Lognormal	(30)
Utility					
Non-bleeding in response	0.86	0.71	1.00	Lognormal	(31)
Mild bleeding in response	0.73	0.54	0.92	Lognormal	(31)
Relapse state without bleeding Non-bleeding in relapse	0.84	0.65	1.00	Lognormal	(31)
Mild bleeding in relapse	0.73	0.54	0.92	Lognormal	(31)
Severe bleeding in relapse	0.45	0.36	0.54	Lognormal	(32)
Cost (CNY)					
Drug cost					
Romiplostim	1,536.00	1,228.80	1,536.00	Gamma	Latest regional procurement price of the imported innovator product
Eltrombopag	2,579.85	2063.88	2,579.85	Gamma	
Hetrombopag	2,774.38	2,219.50	2,774.38	Gamma	Latest regional procurement price of the domestically developed innovative product
Rituximab	1,366.20	1,092.96	1,366.20	Gamma	Latest median bidding price from regional databases
rhTPO	789.00	631.20	789.00	Gamma	
Decitabine	161.50	86.25	1,645.03	Gamma	
Immunoglobulin	561.00	561.00	620.00	Gamma	
Methylprednisolone	38.85	38.85	109.67	Gamma	
Platelet infusion	1,530.00	1,224.00	1,836.00	Gamma	
Disease management cost					
Subcutaneous injection	3.50	2.00	6.00	Gamma	Median prices gathered from medical service price lists of Beijing, Shanghai, Zhejiang, Shandong, Hainan
Ivgtt	7.00	4.50	16.00	Gamma	
Intravenous injection	5.50	4.50	7.00	Gamma	
Bed	50.00	40.00	60.00	Gamma	
Nursing	26.00	20.00	30.00	Gamma	
Follow-up cost					
Diagnosis	38.00	22.00	50.00	Gamma	Median prices gathered from medical service price lists of Beijing, Shanghai, Zhejiang, Shandong, Hainan
Blood test	17.00	15.00	19.00	Gamma	
Liver function test	45.00	31.00	60.00	Gamma	
Discount rate	0.045	0	0.05	Beta	(23)

With regard to the long-term loss-of-response rates of eltrombopag, hetrombopag and romiplostim, the numbers at risk and censoring details required to reproduce the parametric treatment-duration models were unavailable; thus, simplified constant transition probabilities from response to non-response were derived from the mean treatment duration values reported in Lee et al. (24). Assuming an exponential time-to-loss-of-response distribution, the per-cycle transition probability was calculated as *p* = 1 − e^–1/m^, where m is the mean duration of response in 4-week cycles. Due to the absence of long-term comparative data, the relapse probability for hetrombopag was assumed to be equal to that of eltrombopag, based on their similar mechanism of action as thrombopoietin receptor agonists.

Relapse rates of subsequent therapy were estimated by reconstructing individual patient data (IPD) from the relapse-free survival (RFS) curves reported in Chinese clinical trials (25, 26). Parametric survival functions were fitted to the reconstructed data, and the best-fitting distributions were selected for extrapolation to derive long-term relapse rates. Relapse rates for patients who received rituximab and rituximab + rhTPO were obtained from a multicenter randomized controlled trial conducted by Zhou et al. (25). Relapse rates of decitabine were obtained from a prospective multicenter clinical study (26). GetData Graph Digitizer (the version of 2.25) was applied to recreate relapse data based on relapse-free survival curves with the method introduced by Guyot et al. (27). Parametric distributions including exponential, gamma, gompertz, weibull, loglogistic, lognormal were fitted to extrapolate time-depending relapse rates. The selection of the distribution was informed by Akaike Information Criterion (AIC), Bayesian Information Criterion (BIC) and visual inspection. Goodness of fit results were listed in Supplementary Table S3. Supplementary Table S4 showed best-fitting distributions for subsequent treatments and parameters for the simulation of relapse data.

As far as concerned, bleeding rates of ITP patients varied with different health states (28). In reference to published researches (24, 28), when patients stayed in the state of response, they might experience no bleeding or mild bleeding; when patients suffered relapse events, they might experience no bleeding, mild bleeding or severe bleeding. Probabilities of rescue treatments were originated from key clinical trials (18–20).

### Mortality

Mortality rates of response patients were assumed to be natural mortality rates of the general population estimated in the sixth National Census by National Bureau of Statistics (29). For patients who experienced relapse events, the mortality rate was 1.5 times that of the response population as an epidemiological study revealed (30).

### Adverse events

Because none of the included clinical trials reported grade 3–5 adverse events with an incidence exceeding 5% (18–20), adverse event management costs were not incorporated into the model. Given the relatively low incidence of severe adverse events reported in the available evidence and comparable data on severe adverse event-related healthcare utilization were unavailable, the impact of adverse event management costs was expected to be limited.

### Utility inputs

The HRQoL of patients was measured using health utilities. Due to a lack of research on the utility of Chinese ITP patients, utility values were acquired from an HRQoL study which adopted the time-trade off (TTO) utility elicitation technique in England, and a published cost-effectiveness analysis (31, 32). Utility inputs were displayed in Table 1.

### Resource utilization and costs

From the Chinese healthcare system perspective, this analysis merely collected direct medical costs. All costs were updated to 2026 Chinese yuan (CNY).

The acquisition costs of the three TPO-RAs were estimated based on the latest regional procurement prices of their marketed original products in China. For eltrombopag, the price of the imported innovator product was used in the base-case analysis to ensure consistency with the clinical efficacy evidence, which was primarily generated from studies involving the innovator formulation. Costs of other drugs were the latest median bidding prices collected from the regional database. Disease management costs and follow-up costs were median values assembled from the medical service price lists of Shanghai, Zhejiang, Beijing, Shandong, Hainan. Table 1 summarized all model inputs for the calculation of cost-effectiveness outcomes.

### Sensitivity analysis and scenario analysis

The uncertainty surrounding the cost-effectiveness of interventions were exhibited through sensitivity analysis and scenario analysis. Deterministic sensitivity analysis (DSA) was performed to assess the impact of individual parameter uncertainty on model outcomes. Key model inputs, including clinical effectiveness parameters, utility values and treatment costs were varied across plausible ranges derived from confidence intervals or ± 20% of base-case values where data were unavailable. The results were presented using tornado diagrams to identify the parameters with the greatest influence on cost-effectiveness outcomes. Probabilistic sensitivity analysis (PSA) was performed to evaluate the joint uncertainty of all model parameters simultaneously. Probability distributions were assigned to parameters based on their characteristics: beta distributions for probabilities, utilities and discount rate, gamma distributions for costs, and log-normal distribution for relative risk. A Monte Carlo simulation with 1,000 iterations were conducted and the results were shown in the form of cost-effectiveness acceptability curves (CEACs).

Additionally, scenario analyses were undertaken to further investigate the uncertainty arising from different time horizons. To be specific, incremental cost-effectiveness ratios (ICERs) were calculated at 15 and 20 years of simulations to test whether economic results were sensitive to the time horizon.

## Results

### Base-case analysis

In base-case analysis, eltrombopag induced the minimum cost (237,537.56 CNY) while hetrombopag and romiplostim induced 278,867.08 CNY and 336,547.22 CNY, respectively. Romiplostim yielded the maximum health outcomes (13.66 QALYs) while eltrombopag and hetrombopag yielded 13.61 QALYs and 13.62 QALYs, respectively. When compared with eltrombopag, hetrombopag and romiplostim generated an ICER of 2,484,284.17 CNY per QALY and 2,077,773.05 CNY per QALY, respectively. When compared with hetrombopag, romiplostim was associated with an ICER of 1,859,724.27 CNY per QALY. If the WTP was below 2-time of GDP per capita (199,330 CNY), eltrombopag has the potential to demonstrate an economic advantage over hetrombopag and romiplostim. Base-case results were listed in Table 2.

**TABLE 2:** Results of base-case analysis.

Treatment	Total costs (CNY)	Incremental costs (CNY)	Total utilities (QALYs)	Incremental utilities (QALYs)	ICER (vs. eltrombopag)	ICER (vs. hetrombopag)
Eltrombopag	237,537.56	–	13.61	–	–	–
Hetrombopag	278,867.08	41,329.52	13.62	0.02	2,484,284.17	–
Romiplostim	336,547.22	99,009.66	13.66	0.05	2,077,773.05	1,859,724.27

### Sensitivity analysis

**FIGURE 4:**
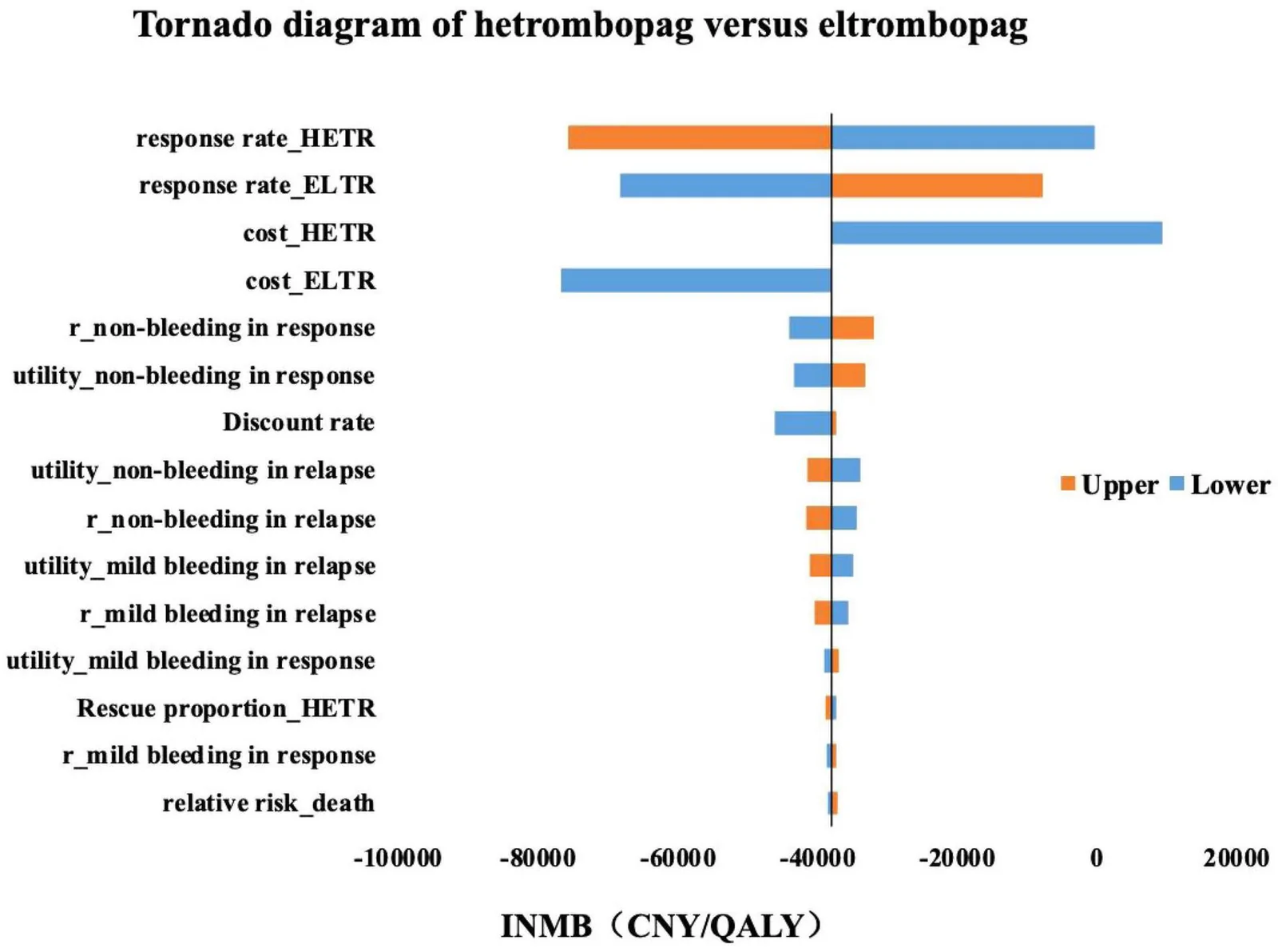
Tornado diagram of hetrombopag compared with eltrombopag. r_non-bleeding in response, non-bleeding rate in responders; r_non-bleeding in relapse, non-bleeding rate in relapsed patients; r_mild bleeding in relapse, mild bleeding rate in relapsed patients; r_mild bleeding in response, mild bleeding rate in responders.

**FIGURE 5:**
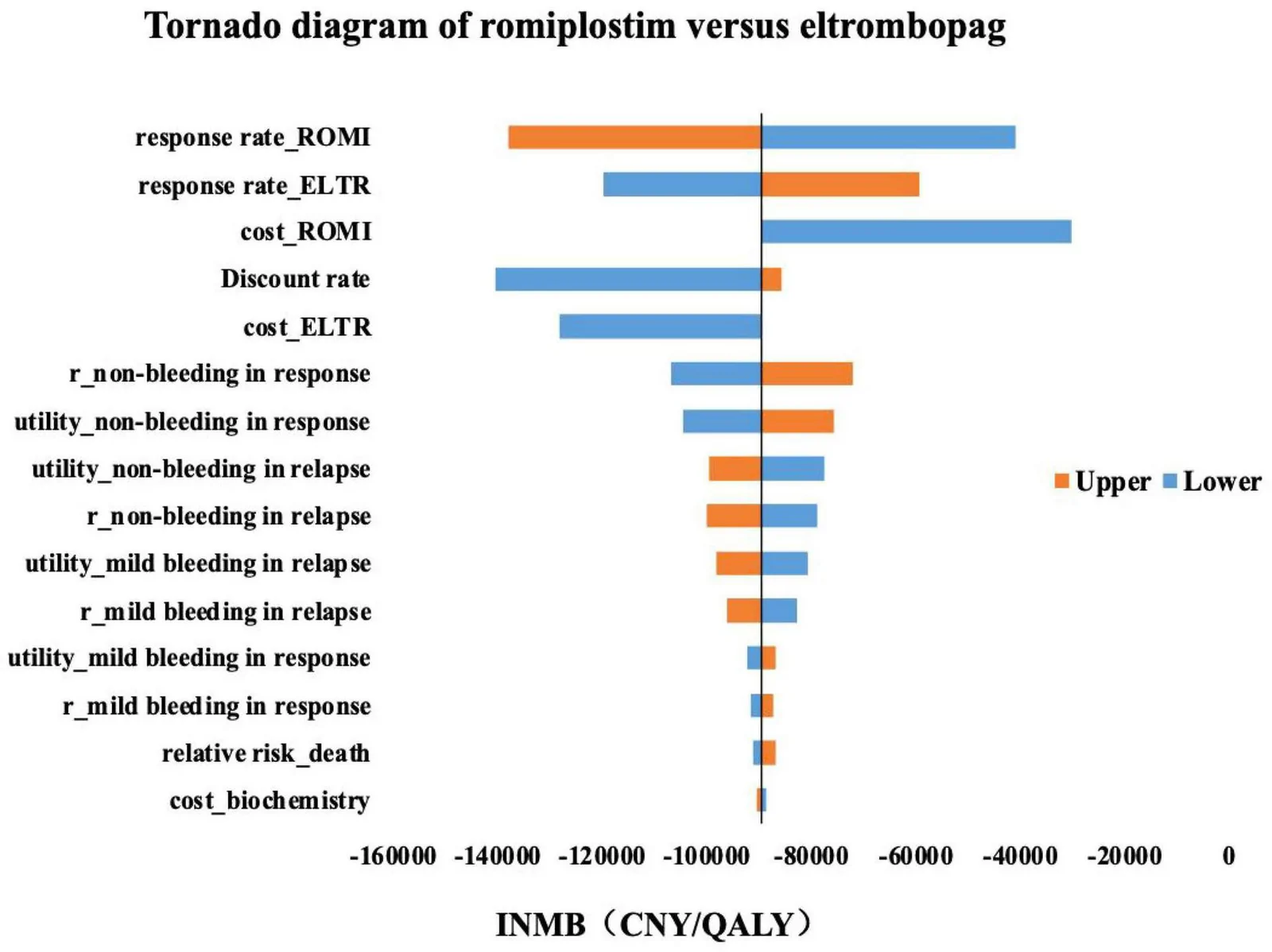
Tornado diagrams of romiplostim compared with eltrombopag. r_non-bleeding in response, non-bleeding rate in responders; r_non-bleeding in relapse, non-bleeding rate in relapsed patients; r_mild bleeding in relapse, mild bleeding rate in relapsed patients; r_mild bleeding in response, mild bleeding rate in responders.

**FIGURE 6:**
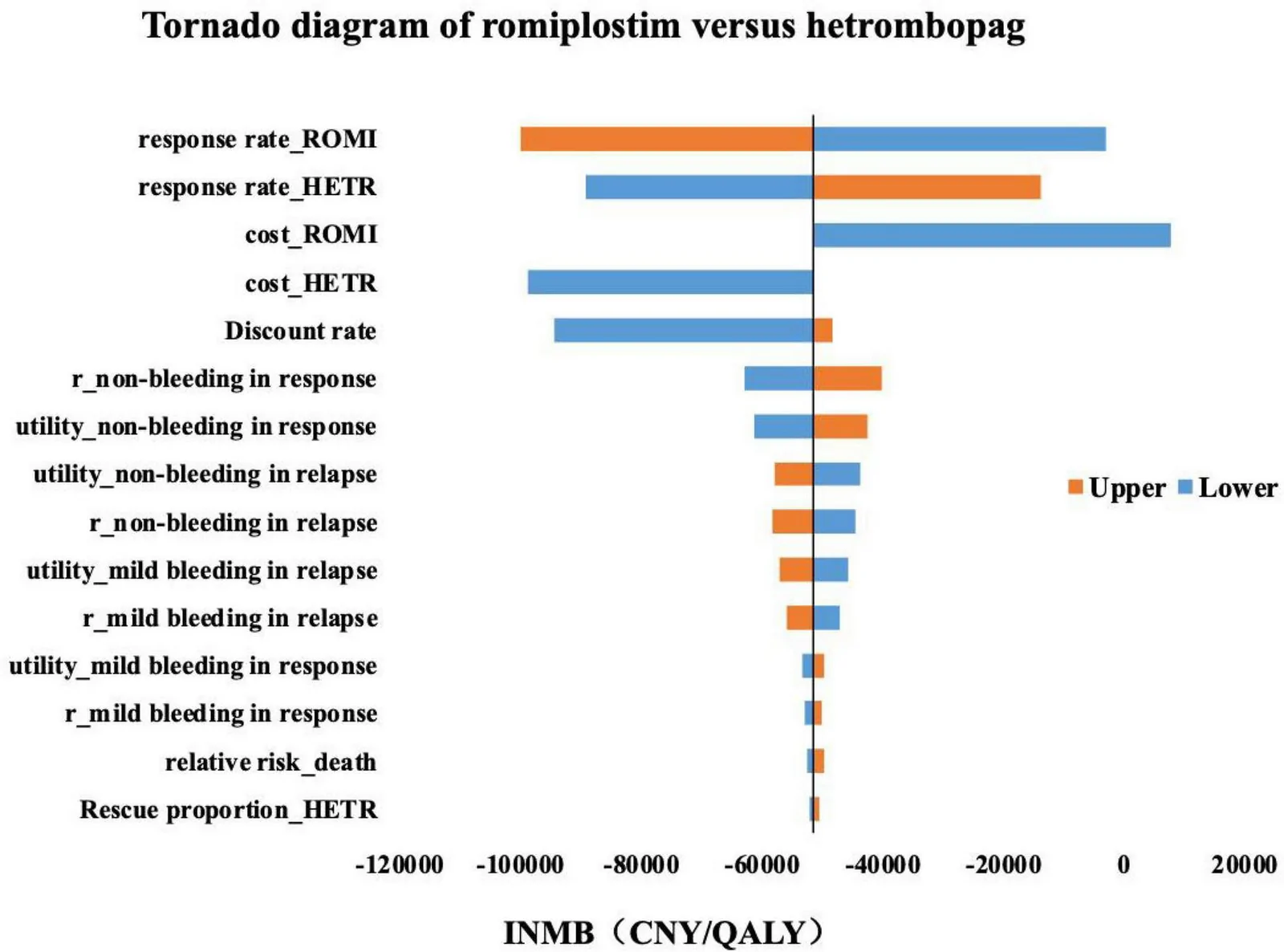
Tornado diagram of romiplostim compared with hetrombopag. r_non-bleeding in response, non-bleeding rate in responders; r_non-bleeding in relapse, non-bleeding rate in relapsed patients; r_mild bleeding in relapse, mild bleeding rate in relapsed patients; r_mild bleeding in response, mild bleeding rate in responders.

**FIGURE 7:**
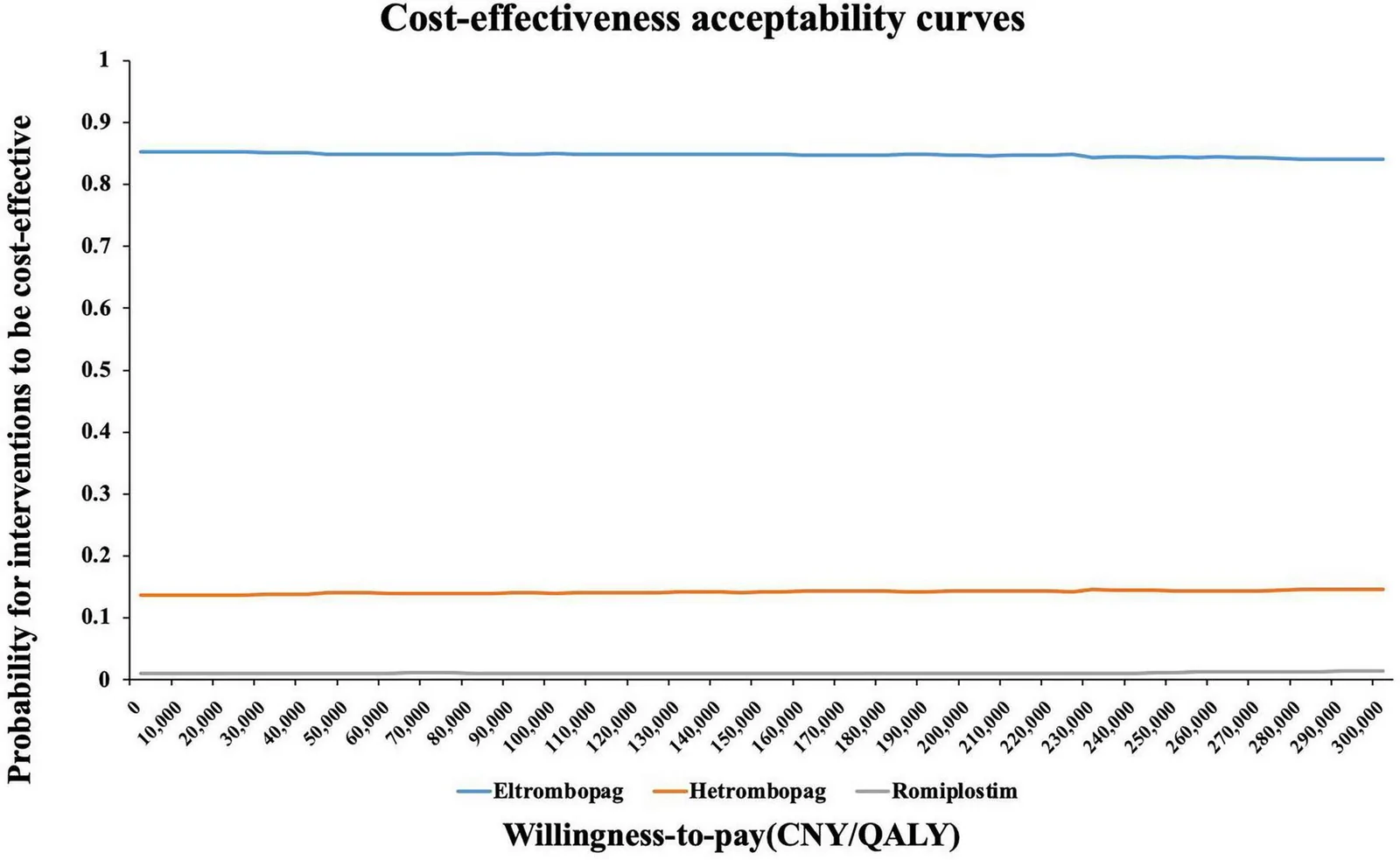
Cost-effectiveness acceptability curves of probabilistic sensitivity analysis.

The deterministic sensitivity analysis showed that the model results were sensitive to response rates, drug acquisition costs and discount rate (Figures 4–6). Variations in the costs of hetrombopag and romiplostim had the greatest impact on the incremental results, and reductions in these costs could potentially lead to a reversal of base-case conclusions. Other parameters, including bleeding rates, utility values had a comparatively smaller influence on the results. Parameters of relapse rates had virtually no effect on base-case conclusions. The probabilistic sensitivity analysis demonstrated that eltrombopag remained the most likely cost-effective option when the WTP threshold was below 2-time GDP per capita (Figure 7).

### Scenario analysis

Varied time horizons were investigated in scenario analyses to further explore the uncertainty of the cost-effectiveness results. When the time frame was shortened to be 20 years, ICERs of hetrombopag and romiplostim compared to eltrombopag were 3,458,288.54 CNY per QALY and 2,629,462.47 CNY per QALY, respectively. When the time frame was shortened to be 15 years, similar conclusions were drawn (Table 3). The results of scenario analysis were consistent with base-case results as a whole.

**TABLE 3:** Results of scenario analyses.

Treatment	Total costs (CNY)	Incremental costs (CNY)	Total utilities (QALYs)	Incremental utilities (QALYs)	ICER (vs. eltrombopag)	ICER (vs. hetrombopag)
20-year simulation						
Eltrombopag	229,793.95	–	11.09	–	–	–
Hetrombopag	270,345.82	40,551.87	11.10	0.01	3,458,288.54	–
Romiplostim	318,624.77	88,830.81	11.13	0.03	2,629,462.47	2,188,837.73
15-year simulation						
Eltrombopag	219,846.03	–	9.23	–	–	–
Hetrombopag	258,878.02	39,031.99	9.24	0.01	4,324,283.34	–
Romiplostim	296,446.93	76,600.90	9.25	0.03	2,928,557.68	2,193,126.82

## Discussion

This study explored the cost-effectiveness of eltrombopag, hetrombopag and romiplostim to treat ITP based on indirect comparisons of clinical data in Chinese population. Base-case analysis revealed that eltrombopag would result in better cost-effective benefit when the WTP threshold of below 2-time GDP per capita was considered. In view of current health decision-making context, eltrombopag was likely to be the cost-effective regimen for Chinese healthcare system. Deterministic sensitivity analysis showed that drug prices were dominant factors which exerted impact on ICERs. Reductions in costs of hetrombopag and romiplostim could potentially change the cost-effectiveness conclusions. Probabilistic sensitivity analysis and scenario analyses confirmed the robustness of base-case results. Although differences in QALYs among the three TPO-RAs were observed, the absolute differences were relatively small. A difference of 0.05 QALYs over a 30-year horizon corresponds to approximately 18 days of perfect health, and its clinical relevance should therefore be interpreted cautiously. Moreover, small variations in QALYs may partly reflect uncertainty associated with long-term model assumptions and parameter inputs, and should be interpreted alongside the sensitivity analyses. These incremental QALYs should be considered together with costs, ICERs, and uncertainty analyses rather than interpreted independently.

Although several second-line options are available for ITP, including splenectomy and rituximab (33), TPO-RAs have become the predominant choice in clinical practice due to their favorable efficacy and tolerability profiles (34). Lee et al. (24) assessed the cost-effectiveness of romiplostim versus eltrombopag and standard of care in the treatment of ITP in Ireland via a lifetime Markov model embedded with a decision tree established, incorporating the health states of response (platelet count ≥ 50 × 109/L), non-response and death. Patients who failed study treatments would switch to receive subsequent therapy involving rituximab, azathioprine, mycophenolatemofetil, cyclosporine according to Irish clinical practice (24). Primary effectiveness outcomes were derived from probability of initial platelet response, average time to initial response and durability of response. This analysis showed that romiplostim was cost-effective with a WTP of €30,000/QALY (24). Allen et al. (35) evaluated the cost-effectiveness of romiplostim and eltrombopag through Markov model populating patient-level data and results showed that eltrombopag was the regimen with better economic value for the UK healthcare system.

Recent economic evaluations have examined subsets of TPO-RAs in the Chinese healthcare setting (36, 37). Luo et al. (36) evaluated the cost-effectiveness of romiplostim versus eltrombopag for Chinese patients with ITP using a decision tree-embedded Markov model with clinical parameter obtained through matching-adjusted indirect comparison (MAIC) based on individual patient data from a phase III RCT in China (38). When 0.5, 0.8, and 1 time GDP per capita were chosen to be the WTP thresholds, romiplostim was not a cost-effective regimen (36). More recently, Jin et al. (37) evaluated the cost-effectiveness of hetrombopag, eltrombopag and avatrombopag among Chinese patients with chronic ITP and reported hetrombopag as the most cost-effective oral TPO-RA under their model assumptions. Compared with these studies, our analysis incorporated romiplostim as an injectable TPO-RA and focused on three TPO-RAs currently reimbursed and clinically used for second-line ITP treatment in China, thereby providing complementary evidence from a broader comparison perspective. Differences in economic outcomes across studies may be explained by variations in comparator selection, model structures, clinical data sources, and cost estimation. Our study further contributes to the economic evidence on TPO-RAs by evaluating oral and injectable options within a unified modeling framework.

This study focused on the second-line treatment for ITP and filled the blank space of head-to-head cost-effectiveness comparison of TPO-RAs covered by NRDL in China. The results served to assist the drug use decision-making in domestic hospitals and better inform the management of TPO-RAs priced through government negotiation for the following reasons. First of all, the model in this article was constructed with recommended model structure and health states (39). Besides, key clinical data of study drugs were obtained from randomized controlled trials in China, ensuring the population representativity. In addition, subsequent regimens were considered to mimic realistic treatment procedure in accordance with the recommendation of current clinical guideline for ITP in China (11). In this analysis, the cost of eltrombopag was based on the price of the imported innovator product. Despite that lower-priced generic eltrombopag products may further improve the cost-effectiveness of eltrombopag in clinical practice, the present analysis focused on the imported innovator drug to maintain consistency with the available efficacy evidence.

The study results should be interpreted with caution because there existed limitation of clinical data. Due to a lack of head-to-head clinical studies on eltrombopag, hetrombopag and romiplostim, an indirect comparison was undertaken in this article to obtain response rates. Since no time-to-relapse curves were reported in clinical studies on eltrombopag, hetrombopag and romiplostim, constant relapse rates of study drugs were assumed based on calculations. However, deterministic sensitivity analysis confirmed that relapse rates did not materially affect the economic results, indicating that this limitation is unlikely to bias the overall conclusions. Besides, this analysis did not fully capture treatment modifications that may occur in real-world practice, including dose reductions and treatment discontinuations, which may influence drug costs and health outcomes. Furthermore, adverse event management costs and potential differences in safety profiles among TPO-RAs were not explicitly incorporated due to limited evidence regarding their impact on healthcare utilization and comparable long-term safety data across treatments. Future real-world studies evaluating treatment patterns and long-term safety outcomes may help improve economic evaluations of TPO-RAs. Finally, health utility values were obtained from relevant study based on foreign population because HRQoL studies for Chinese ITP populations were not available. This study can be improved if direct effectiveness comparison of TPO-RAs, long-term follow-up researches as well as real-world studies were published in the future.

## Conclusion

The results of this cost-effectiveness analysis showed that eltrombopag was likely to be cost-effective compared with hetrombopag and romiplostim in treating patients with ITP who failed first-line therapy. If the prices of hetrombopag and romiplostim were reduced, their cost-effectiveness would be improved. These findings provide useful economic evidence for clinicians and decision-makers when considering second-line treatment options for ITP and evaluating the value of TPO-RAs in China. Further long-term clinical studies are needed to reduce uncertainty in future economic evaluations.

## Data Availability

The original contributions presented in the study are included in the article/Supplementary material, further inquiries can be directed to the corresponding author.
