# Supplementary material for: Cost-effectiveness analysis of eltrombopag, hetrombopag and romiplostim for second-line treatment of primary immune thrombocytopenia in China
Source: Front Med (Lausanne). 2026 Aug 24;13:1916651. doi: 10.3389/fmed.2026.1916651 (PMC13546990; doi:10.3389/fmed.2026.1916651)
Supplement: Supplementary file 1 [file Supplementary_file_1.docx]

**Cost-effectiveness Analysis of Eltrombopag, Hetrombopag and Romiplostim for Second-line Treatment of Primary Immune Thrombocytopenia in China**

**Table S1 Basic information of key RCTs**

| **Study ID** | **Group** | **Indica**  **-tions** | **Patient source** | **Interventions** | **Time frame**  **(weeks)** | **Sample size** | **Average age** | **Proportion of males (%)** | **Response rates**  **(%)** | **Proportion of rescue events**  **(%)** |
| --- | --- | --- | --- | --- | --- | --- | --- | --- | --- | --- |
| Yang2017 | Treatment group | Chronic  immune thromboc-ytopenia | China | Eltrombopag | 6 | 104 | 44.7 | 26.0 | 57.7 | 8.7 |
|  | Control group |  |  | Placebo | 6 | 50 | 41.3 | 21.6 | 6.0 | 34 |
| Mei2021 | Treatment group | Primary immune thromboc-ytopenia | China | Hetrombopag | 8 | 171 | 38 | 27.4 | 64.3 | 9.9 |
|  | Control group |  |  | Placebo | 8 | 85 | 42 | 29.4 | 5.9 | 37.6 |
| Zhou2023 | Treatment group | Persistent or chronic primary immune thromboc-ytopenia | China | Romiplostim | 6 | 140 | 42.1 | 33.1 | 50.0 | 1.4 |
|  | Control group |  |  | Placebo | 6 | 46 | 39.7 | 27.5 | 2.2 | 2.2 |

**Figure S1 Relative treatment effects versus placebo from indirect comparison**

**Table S2 Estimated absolute response rates using pooled placebo as anchor**

| **Treatment** | **Pooled placebo response rate** | **OR versus pooled placebo** | **Estimated absolute response rate** |
| --- | --- | --- | --- |
| Eltrombopag | 0.05 | 21.36 | 0.53 |
| Hetrombopag |  | 28.85 | 0.60 |
| Romiplostim |  | 45.00 | 0.70 |

*Absolute response rates were derived by applying treatment-specific odds ratios versus placebo to the pooled placebo response rate using the formula: p = p_0_ × OR / (1 - p_0_ + p_0_ × OR), where p_0_ denotes the pooled placebo response rate.*

**Table S3 Goodness of fit results of time-to-relapse survival fitting**

|  | **Regimen** | **Exponential** | **Gamma** | **Gompertz** | **Weibull** | **Loglogistic** | **Lognormal** |
| --- | --- | --- | --- | --- | --- | --- | --- |
| AIC | RTX | 141.607 | 143.441 | 139.605 | 142.981 | 139.246 | 138.075 |
|  | RTX+rhTPO | 288.768 | 290.681 | 287.672 | 290.394 | 287.087 | 284.935 |
|  | Decitabine | 78.437 | 76.887 | 73.547 | 75.938 | 77.371 | 78.457 |
| BIC | RTX | 142.903 | 146.033 | 142.196 | 145.573 | 141.838 | 140.666 |
|  | RTX+rhTPO | 290.878 | 294.902 | 291.894 | 294.616 | 291.309 | 289.157 |
|  | Decitabine | 79.573 | 79.158 | 75.818 | 78.209 | 79.642 | 80.728 |

**Table S4 Best-fitting distribution and survival fitting parameters**

| **Regimen** | **distribution** | **lambda** | **sigma** |
| --- | --- | --- | --- |
| RTX | lognormal | 2.13 | 1.31 |
| RTX+rhTPO | lognormal | 2.64 | 1.42 |
| Decitabine | exponential | 0.02 | / |

**Reference**

[1] Yang R, Li J, Jin J, Huang M, Yu Z, Xu X, et al. Multicentre, randomised phase III study of the efficacy and safety of eltrombopag in Chinese patients with chronic immune thrombocytopenia. Br J Haematol. 2017;176(1):101-110.

[2] Mei H, Liu X, Li Y, Zhou H, Feng Y, Gao G, et al. A multicenter, randomized phase III trial of hetrombopag: a novel thrombopoietin receptor agonist for the treatment of immune thrombocytopenia. J Hematol Oncol. 2021;14(1):37.

[3] Zhou H, Zhou J, Wu D, Ma L, Du X, Niu T, et al. Romiplostim in primary immune thrombocytopenia that is persistent or chronic: phase III multicenter, randomized, placebo-controlled clinical trial in China. Res Pract Thromb Haemost. 2023;7(5),100192.
